# Application of Quality by Design in the Development of Hydrogen Sulfide Donor Loaded Polymeric Microparticles

**DOI:** 10.1208/s12249-024-02840-8

**Published:** 2024-06-07

**Authors:** Anjali Rai, Susmit Mhatre, Cole Chandler, Catherine Opere, Somnath Singh

**Affiliations:** 1 Department of Pharmacy Sciences, School of Pharmacy and Health Professions, Creighton University, 2500 California Plaza, Omaha, NE 68178, USA; 2 Department of Biology, College of Arts and Sciences, Creighton University, Omaha, NE 68178, USA

**Keywords:** design of experiments, glaucoma, hydrogen sulfide, polymeric microparticles, quality-by-Design, sustained release

## Abstract

Hydrogen sulfide (H_2_S) is a multifaceted gasotransmitter molecule which has potential applications in many pathological conditions including in lowering intraocular pressure and providing retinal neuroprotection. However, its unique physicochemical properties pose several challenges for developing its efficient and safe delivery method system. This study aims to overcome challenges related to H_2_S toxicity, gaseous nature, and narrow therapeutic concentrations range by developing polymeric microparticles to sustain the release of H_2_S for an extended period. Various formulation parameters and their interactions are quantitatively identified using Quality-by-Design (QbD) approach to optimize the microparticle-based H_2_S donor (HSD) delivery system. Microparticles were prepared using a solvent-evaporation coacervation process by using polycaprolactone (PCL), soy lecithin, dichloromethane, Na_2_S.9H_2_O, and silicone oil as polymer, surfactant, solvent, HSD, and dispersion medium, respectively. The microparticles were characterized for size, size distribution, entrapment efficiency, and H_2_S release profile. A Main Effects Screening (MES) and a Response Surface Design (RSD) model-based Box-Behnken Design (BBD) was developed to establish the relationship between critical process parameters (CPPs) and critical quality attributes (CQAs) qualitatively and quantitatively. The MES model identified polymer to drug ratio and dispersion medium quantity as significant CPPs among others, while the RSD model established their quantitative relationship. Finally, the target product performance was validated by comparing predicted and experimental outcomes. The QbD approach helped in achieving overall desired microparticle characteristics with fewer trials and provided a mathematical relationship between the CPPs and the CQAs useful for further manipulation and optimization of release profile up to at least 30 days.

## Introduction

Hydrogen sulfide (H_2_S) has been known as a toxic gas for several centuries. The research on H_2_S mainly focused on understanding its toxicological profile until the late twentieth century when its pharmacological functions were discovered [1]. In the late 1980’s and 1990’s, pioneering work by several research groups led to the discovery of H_2_S as an endogenous gasotransmitter with pharmacological and physiological roles in the mammalian cells [1–3]. Subsequent studies have also reported its roles in cytoprotection [4–8], neuroprotection [9, 10], smooth muscle relaxation [11, 12], vasorelaxation and regulation of blood pressure [12–18], anti-inflammation [5, 19–24], cellular respiration [25–29], etc. Owing to its critical role in mammalian pathophysiology, H_2_S has found applications in several conditions as a therapeutic molecule [30–32].

Our group is interested in the application of H_2_S in glaucoma, an ocular neurodegenerative disorder. Glaucoma is characterized by progressive loss of retinal ganglion cells (RGC) and cupping (enlargement of the cup to disc ratio) of the optic nerve head, leading to visual defects and loss of vision [33]. Increase in aqueous humor production and/or decrease in its outflow are clinically modifiable risk factor in glaucoma that result in elevated intraocular pressures (IOP). The increase in IOP causes the lamina cribrosa to compress, distort and remodel, ultimately causing the apoptosis of RGCs [34] resulting in vision loss. Hence, clinically available glaucoma drugs aim to restore the IOP to a healthy range of 10–22 mmHg via different mechanisms [35]. However, glaucomatous damage has also been reported in normotensive patients [36]. Moreover, the currently used glaucoma drugs are also reported to exhibit many adverse effects [37–41]. Thus, there is a need of a drug that not only restores normal IOP but also prevents the underlying RGC loss.

We are interested in investigating H_2_S as a potential candidate to provide both ocular hypotensive and retinal neuroprotective effects. We have previously shown that H_2_S donors (HSD) can successfully lower IOP in normotensive rabbits *in vivo* [42], modulate aqueous humor dynamics in porcine ocular tissues *ex vivo* [43], mitigate peroxide induced oxidative stress in bovine retina *ex vivo* [44], and show high bioavailability in bovine retina tissues, *ex vivo* [45]. These studies support the potential of H_2_S as a superior candidate for glaucoma.

Despite its pharmacological actions, the clinical translation of H_2_S in glaucoma has several challenges. Its gaseous nature at physiologically relevant temperatures and pressure, inherent toxicity, therapeutic activity at low concentrations, and instability in aqueous solutions are deterrents to deliver H_2_S topically. Hence, a delivery system that controls the release of H_2_S is necessary to translate the pharmacological potential of this gasotransmitter. We have previously discussed in detail about the challenges and strategies of HSD delivery with relevant delivery examples including those with polymeric particles [46]. From an ocular delivery standpoint, eye drops and ointments are the most used dose forms in topical ocular delivery owing to their patient acceptability and non-invasive delivery route. Nevertheless, as eye drops often utilize and aqueous base, they are not suitable for active molecules that are water unstable, such as HSD. The bioavailability of the finished product can be significantly reduced as a result of the quick release of H_2_S gas caused by the instability of HSD in an aqueous environment [47, 48] due to the presence of dissolved oxygen. In contrast, HSD can be incorporated into ointments using non-aqueous bases. However, ointments require repeated applications owing to lower drug-loading capacity. They can cause obscured vision due to its greasy and sticky characteristic resulting in poor patient compliance [49].

Thus, to prevent toxicity and other adverse effects, a clinically relevant H_2_S delivery system should provide therapeutically appropriate bioavailability in the anterior and posterior parts of the eye at a low potential therapeutic concentration range (100 nM—100 μM) [50] over an extended period of time. We propose an injectable route for the delivery of HSD. As compared to intravitreal injection, a subconjunctival route will offer advantages such as being less invasive, administration of *in situ* gelling solutions, dual delivery targeted to anterior and posterior segments, slow and sustained release possible, and overcoming the conjunctival-corneal barrier. A sustained release HSD formulation will ensure the recommended dosing frequency of once in four or more weeks.

We hypothesize that polymeric microparticles of HSD using a slow degrading hydrophobic polymer can sustain the release of HSD to achieve ideal therapeutic outcomes [51]. The rationale for developing microparticle, instead of nanoparticle, was its ability to entrap higher drug load, thereby sustaining a slower rate of drug-release for an extended period. An optimal drug load and particle size are required to ensure easy injectability and required bioavailability of H_2_S in the posterior segment of the eye.

Traditional trial and error-based drug delivery development approaches are not appropriate and economically feasible because HSDs are very expensive [52] with narrow therapeutic window in nmolar range [49]. Therefore, the Quality-by-Design (QbD)-based approach was used in preparing HSD-loaded microparticles to minimize number of formulations needed to develop a therapeutically relevant optimized formulation.

The QbD is a predictive statistical approach to achieve a desired quality in a product or experimental outcome by incorporating the quality attributes into product development process. In the conventional approach, termed quality-by-testing (QbT), quality of a product is evaluated by testing the final product performance by manipulating one quality attribute at a time. This approach cannot evaluate how all the quality attributes can influence the product performance while working simultaneously. Thus, the QbT approach is limited because the final quality cannot be guaranteed as it involves back-and-forth process of using the available data and designing new experiments [53], utilizes a lot of resources, and time. The QbD approach is superior to the QbT approach since it offers a time and cost-effective way to achieve a better quality of the product. Therefore, in this study we employed a QbD approach to develop polymeric microparticles loaded with a model HSD, sodium sulfide (Na_2_S.9H_2_O). The primary focus of this manuscript is harnessing the efficiency of QbD approach in achieving ideal attributes of the HSD microparticles for application in glaucoma.

## Material and Method

### Materials

Dichloromethane (Lot # MKCP0009) and silicone oil (10 cSt; lot # MKCR1941, 20 cSt; lot # MKCR0451, and 100 cSt; lot # MKCS1157) were purchased from Sigma Aldrich, MO, USA. n-Hexane (Lot # G28N770) and soy lecithin (lot # Y01C015) bought from Alfa Aesar, MA, USA. Polycaprolactone (lot # MKCC5704) and sodium sulfide (lot # A0412804) were purchased form Aldrich, MO, USA and Acros Organics, NJ, USA, respectively. These chemicals were used as received. All other reagents used were of ACS quality.

### Methods

#### Preparation of Polymeric Microparticles

**Fig. 1:**
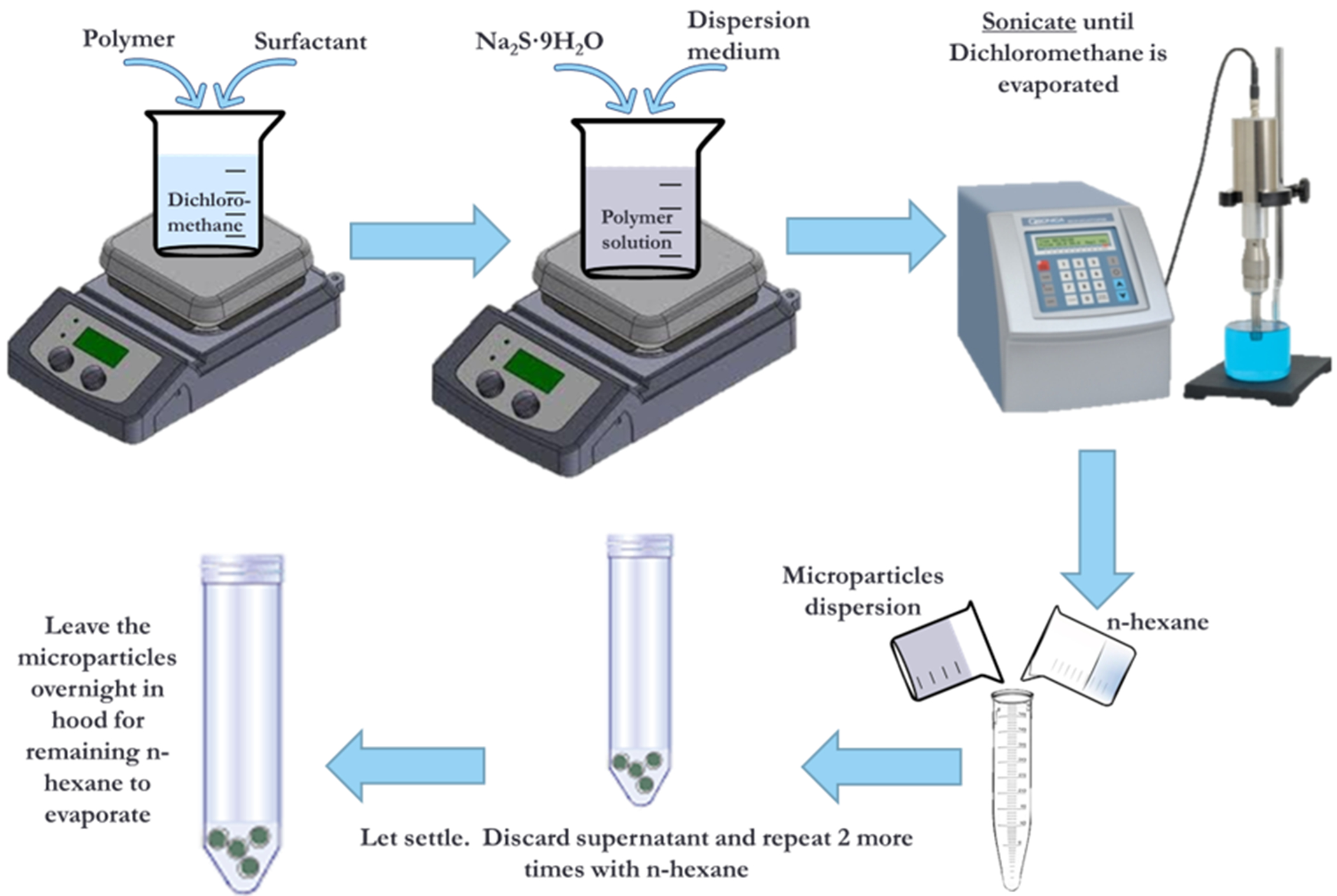
A schematic representation of the steps involved in the preparation polymeric microparticle loaded with sodium sulfide

The schematic representation of polymeric microparticle preparation is depicted in Fig. 1. Briefly, a polymeric solution was prepared by adding a specific amount of polymer (polycaprolactone; MW 14,000) and surfactant (soy lecithin) to 10 mL dichloromethane. It was subjected to mixing using a vortex until a clear solution was obtained. A specific amount of Na_2_S.9H_2_O, crushed using a mortar pestle, and dispersion medium (silicone oil) was added to a centrifuge tube, mixed using a vortex, and added to the polymer solution. This drug-polymer solution was sonicated using a Sonic Dismembranator (Model 700, Fisher Scientific, NH, USA) at specific sonication energy and time until the dichloromethane was evaporated, leaving coacervates of polymeric microparticles at the bottom of the tube dispersed in silicone oil. To remove silicone oil, n-hexane was added, and the mixtures were vortexed (for 30 s) and allowed to stand until a clear solution of polymeric precipitates was obtained. The supernatant containing silicone oil dissolved in n-hexane was carefully discarded. This step of washing with n-hexane was repeated twice. Finally, the precipitated microparticles were left open for 24 h in a fume hood at 100fpm to remove any leftover n-hexane in the formulation.

### Characterization of Polymeric Microparticles

#### Determination of Particle Size

The prepared microparticles were analyzed for particle size and particle size distribution using Mastersizer 2000 (Malvern Instruments, PA, USA). The percent (%) obscuration limit was set between 0.5% and 5%. A small quantity of the prepared microparticles were added to the Mastersizer and three measurements were made. The average of the three measurements were represented by the software.

#### Determination of Entrapment Efficiency

To determine the percentage entrapment efficiency (%EE), 100 mg of microparticles were dispersed in argonated distilled water in a tube to dissolve any un-entrapped HSD adsorbed on the surface of microparticles. Next, the microparticles were separated from the water phase and 10 mL dichloromethane was added to the tube to dissolve the polymer and surfactant. Ten mL argonated distilled water was added to the same tube and vortexed (for 60 s) to dissolve the entrapped HSD in the water phase. This biphasic mixture was centrifuged at 1,000 rpm at 4 °C for 10 min. The water phase appeared at the top of the dichloromethane phase. An aliquot from the water phase (supernatant) was used to analyze the H_2_S content using the ISO-H_2_S-100 electrode (World Precision Instruments, FL, USA). Entrapment efficiency was calculated using the formula:

(1) $$\%EE=\frac{\mathrm{Amount of}H_{2}S\;\mathrm{in the supernatant}}{\mathrm{Amount of HSD used to prepare microparticles}}\times 100$$

#### Determination of H_2_S Release from Microparticles

To determine the release profile of H_2_S from polymeric microparticles, 100 mg of microparticles were suspended in 10 mL isotonic PBS buffer (**pH 7.2**) in a 20 mL glass vial. The glass vial was placed in a rotating shaker bath set at 37 °C at 30 rpm. A specific amount of sample (20–100 μL) was collected at specific time points (0, time points) and replaced with an equal volume of fresh PBS buffer pre-warmed at 37 °C. Each sample was evaluated for H_2_S content using the WPI ISO-H_2_S-100 electrode (World Precision Instruments, FL, USA) as reported by us earlier [53]. Specifically, the WPI ISO-100-H_2_S sensor was immersed and polarized in 20 mL of 0.1 M PBS (pH 7.2) for 12 h. The polarized sensor was connected to the free radical analyser (TBR4100) using microsensor cable (WPI, Cat. # 91580) and immersed in a vial containing 20 mL 0.05 M PBS (pH 7.2) placed on a magnetic stirring plate. The free radical analyzer was set to send current of 10 nA at the poise voltage of + 150 mV to the electrode. Samples were added to the electrode environment which resulted in the increased measurement of current by the analyzer. This increase in current corresponded to the concentration of H_2_S in the sample.

The amount of H_2_S released from the microparticle was determined in 20 mL of releasing media. Any error, due to volatile nature of H_2_S, in such concentration measurement would not be significant because the theoretically possible maximum H_2_S released (0.21 mmoles) was less than its solubility in 20 mL of releasing media. The aqueous solubility of H_2_S is 3.9 g/L (i.e. 115 mmoles/L) at 20 °C [54]. Therefore, 20 mL of releasing media can dissolve up to 2.3 mmoles of H_2_S whereas, 50 mg HSD (Na_2_S.9H_2_O) can generate only up to 0.21 mmoles of H_2_S. Moreover, the possible error in concentration measurement was further nullified by using pH 7.2 at which the electrode is more sensitive to dissolved H_2_S [55].

## Critical Consideration in the Application of QbD Approach

### Determination of Critical Quality Attributes (CQAs) and Asssessment of Risk

The determination of CQAs and critical process parameters (CPPs) are paramount for optimizing the process parameters and producing the final products capable of exhibiting desired outcomes. The CQAs were identified, for this study, based on the properties of polymeric microparticles in ocular delivery of a potential gaseous therapeutics targeting glaucoma as discussed in section titled “Defining CQAs of an optimal product” below. We designed four Ishikawa diagrams (fishbone diagrams or cause-effect diagrams) to evaluate the potential risks affecting product quality. The Ishikawa diagrams were constructed by carefully evaluating the formulation and process parameters important in deciding the characteristics of the final product.

### Designing a Main Effects Screening (MES) QbD

#### Defining CQAs of an Optimal Product

The CQAs can be determined by considering ideal profile of target product for maximum therapeutic outcomes. The size of the microparticles to be injected into the eye sub-conjunctivally, must be syringeable and injectable through a needle of 22G or greater to ensure patient comfort and ocular safety [56]. The internal and outer diameter of a 22G needles [56, 57] are 0.413 μm and 0.718 μm, respectively. However, smaller the outer diameter, more convenient will be the subconjunctival injection. Therefore, this study plans to use a 30 G needle in future *in vivo* study which outer diameter is 0.312 μm. However, its internal diameter is 159 μm [58] which in turn means the particle size should be less than 159 μm.

The particle size distribution is also critical to ensure size homogeneity. Hence, particle size and particle size distribution were identified as CQAs. The microparticles must entrap HSD to ensure sustained release of H_2_S while minimize burst release associated with drug adsorbed on the surface of the particles. The efficiency of entrapment is thus another CQA in this study. Lastly, an ideal glaucoma drug delivery system should be able to sustain the release of H_2_S for at least 28 days according to guidelines set by the FDA for once-a-month administration frequency [59, 60]. The longer the drug release period, the better patient compliance, owing to the decreased requirement of dosing frequency. Hence, the drug release time is the fourth CQA identified in this study.

#### Defining Critical Process Parameters (CPPs)

For the process described in section “Preparation of Polymeric Microparticles”, there were several important process parameters such as the amount of the polymer (polycaprolactone), surfactant (soy lecithin), the drug (HSD), dichloromethane, n-hexane, the quantity and viscosity of silicone oil, sonication time and energy. However, from a critical analysis of the process and factors in Ishikawa diagrams, we can exempt the variables such as the amount of dichloromethane, amount of hexane, and settling time since these variables do not affect the final product but rather serve other roles in the process. Conversely, there were several other key process parameters identified for this study such as polymer to drug ratio, polymer to surfactant ratio, the quantity and viscosity of the dispersion medium, sonication time and energy which are reported to significantly influence the final product outcomes. These key parameters were fed into the MES and tested by surface response models. If the fed parameters are found to have significant effect on the desired product quality, they are considered as CPPs.

## Building the MES Model

The MES Model was developed using DoE (Design of Experiment) tab in JMP Pro (16.1), a statistical suite by SAS (Statistical Analysis System) institute (100 SAS Campus Drive, Cary, NC 27513). The MES is a classical 2-level screening design. Particle size, particle size distribution, entrapment efficiency, and drug release were set as responses. The goal for size distribution was to minimize, while for other three responses was to maximize. The upper limit value of 159 μm for particle size, 100% for entrapment efficiency, and set to maximum time for drug release, while the upper limit value for size distribution was set to 0.5 indicating uniform sized particles. Polymer to surfactant ratio with values of 5 to 10, polymer to drug ratio with value of 2 to 20, silicone oil viscosity in categorical values of 10, 20, and 100 cSt, sonication energy in categorical values of 20, 40, 60, 80, and 100 amplitudes, sonication time in categorical values of 30 and 60 min, and dispersion medium with a lower value of 3 g and a higher value of 8 g were identified as the key factors for the process parameters. Using these responses and factors, a custom number of trials of 12 was set. The design had a chi-square efficiency of 95.52%. For formulating the combinations in this design experimentally, the run order was randomized to eliminate series bias. When the formulations were prepared and characterized using techniques described in sections titled “Preparation of polymeric microparticle” and “Characterization of polymeric microparticles”, the evaluations were fed back into JMP, and the model was run with particle size, size distribution, entrapment efficiency, and drug release as variables. All the process parameters were assigned as x-variables. Two-factor interactions were allowed between the parameters to investigate their simultaneous effect on CQAs. The statistical approach of standard least squares with an emphasis on effect screening was adopted. After running this model, factors with statistically significant values (p < 0.05) were identified as CPPs and taken forward in the second-generation response surface design. The factors that had p < 0.05 significance was optimized using the prediction profile generated based on the model. These factors were kept constant for the response surface design. The values of these factors were auto assigned based on desirability set when defining the factors.

### Designing a Response Surface QbD

#### Defining Critical Quality Attributes (CQAs)

The CQAs were set the same as described earlier for the MES design.

#### Defining Critical Process Parameters (CPPs)

The RSD produces a quantitative higher degree relationship between the factors and the responses. It is pertinent to note that only continuous variables can be declared as factors in RSD. Based on results from the MES design, the factors that had a statistically significant effect on the responses (CQAs) p < 0.05 were used in RSD. For the parameters that did not significantly affect the responses, the values for maximum desirability, as predicted from the MES design, were set constant in all the formulations prepared during the RSD design.

### Building the RSD Model

The RSD Model was developed using JMP Pro (16.1) a Box-Behnken design (BBD) with three orthogonal blocks having a block size of 9 points each. The values for CQAs and significant CPPs were defined as in MES model. The blocks control and account for potential sources of variability that might affect results while using the BBD. For formulating these designs experimentally, the run order was randomized to eliminate series bias. When the formulations were prepared and characterized using techniques described in Methods section*,* the experimental outcomes were fed back into JMP, and the model was run with particle size, particle size distribution, entrapment efficiency, and drug release as variables. Analysis was carried similar to that in the MES model.

### Validation of RSD Model

To validate the RSD, the model-predicted values of all factors were used in the formulation of microparticles experimentally (n = 3). These microparticles were characterized for particle size, particle size distribution, entrapment efficiency, and drug release. The responses were compared with model-predicted values for the same responses.

## Results and Discussion

### Defining the CQAs and Risk Assessment

**Fig. 2:**
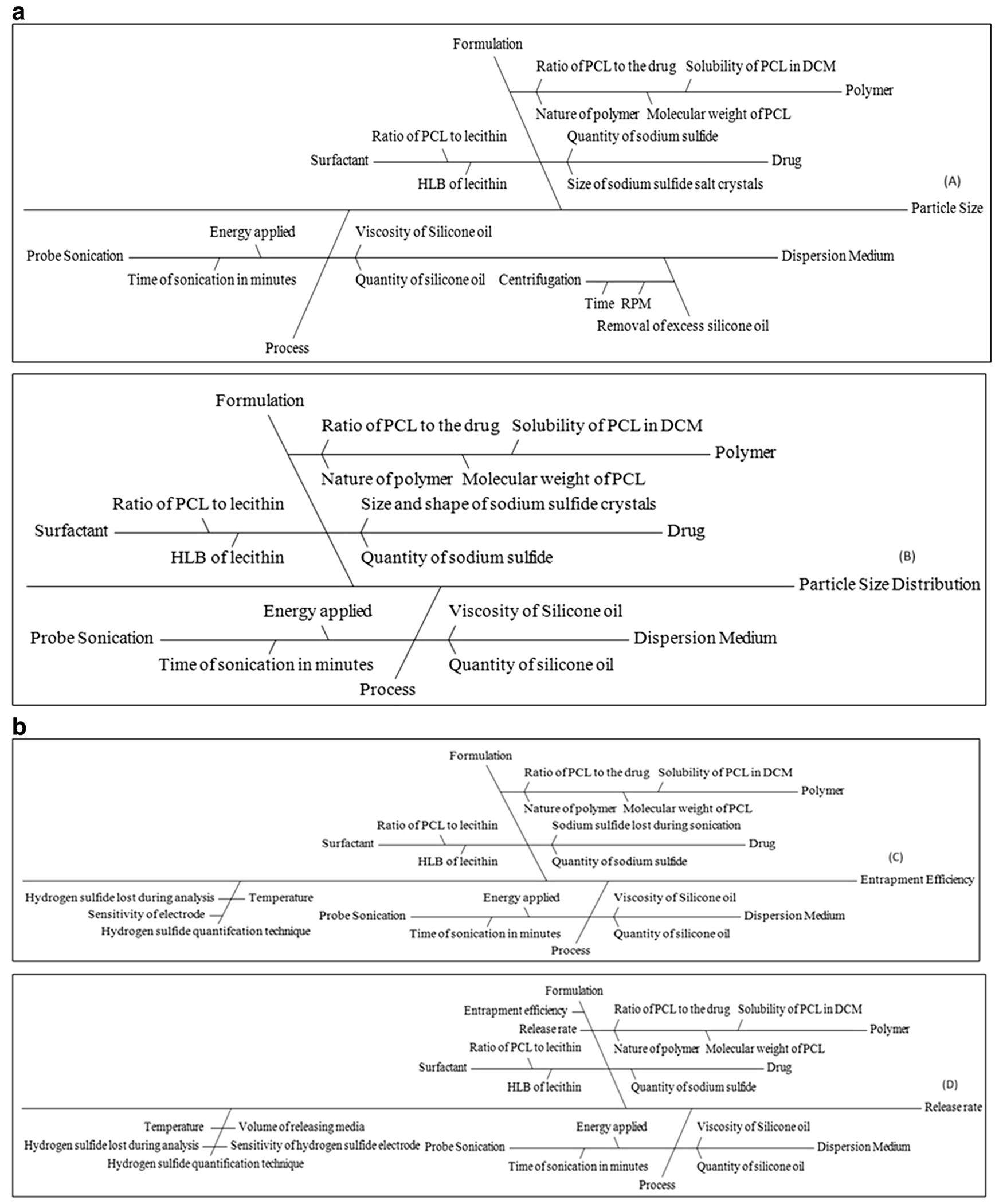
Ishikawa diagrams representing **a** the formulation parameters potentially affecting (A) particle size and (B) particle size distribution; and **b** process parameters affecting (C) entrapment efficiency and (D) release rate

The CQAs are properties (e.g. the correct amount of excipients and drugs) which can influence Quality Target Product Profile (QTPP), thereby, overall final product performance outcomes [61, 62]. The QTPP is mainly concerned with those product properties related with patient clinical outcomes [63] Conversely, risk assessment is identifying what can go wrong and its likelihood, and its impact on CQAs [64]. Here, preparation of the microparticle loaded with HSD is the product expected to release H_2_S at a sustained rate for an extended period. Risk assessment was performed to identify the CQAs that practically affect QTPP responsible for controlling overall quality and safety of the product. Using the Ishikawa diagrams (Fig. 2), particle size, particle size distribution, entrapment efficiency, and release duration were identified as the CQAs in this study.

Based on the safety data sheets, soy lecithin is not considered to be toxic to the eyes. However, it may cause slight irritation to the skin [65, 66]. Similarly, polycaprolactone (PCL) safety data sheet indicates its non-toxicity [67]. Its nanofibers implanted under the conjunctiva and in the corneal stroma of rabbit as patch graft showed minimum inflammation in various ocular tissues such as in the cornea, conjunctiva, and anterior chamber. Additionally, merger between the host tissue and PCL fibers was found with minimal tissue reaction on pathological examination [68] whereas another study found it biocompatible [69]. Furthermore, hexane is not expected to be present in the final product because it is a highly volatile organic compound as indicated by its evaporation rate of 15.8 [70]. Therefore, it would be easily lost to environment during drying the microparticles in the hood (air flow rate = 100 fpm) overnight. However, our future study does include determining the residual hexane in the formulation by using NMR and gas chromatography techniques. Moreover, our future study also includes investigating safety and toxicity profile of the final developed formulation and its components using MTT and TUNEL assays, proteomics, and microscopic techniques.

### Main Effects Screening (MES) QbD

Main Effects Screening was employed in this study to screen the key process parameters and identify the CPPS that affect the CQAs in the formulation of microparticles. Using JMP, the CQAs (responses) and the key process parameters (factors) were fed to the MES design, and the program produced a design consisting of 12 trials formulations which parameters are shown in Table I. These 12 formulations suggested by the model were prepared and characterized for particle size, particle size distribution, release time, and entrapment efficiency, and the results obtained is depicted in Table II.

As seen in Table II, the particle size range for microparticles was 51–141 μm, size distribution from 1.07–3.35, entrapment efficiency from 2 to 47%, and drug release time from burst release (0 h) to 12 h. When the experimental outcomes were input into the MES design and analyzed using the least squares method, a detailed report on the effect of the process parameters on the CQAs was obtained. The MES QbD identified polymer-drug ratio (p < 0.01), dispersion medium quantity (p < 0.01), dispersion medium viscosity (p < 0.05), sonication time (p < 0.01), and sonication energy (p < 0.05) as significant CPPs affecting CQAs (Table III). It was interesting to note that polymer-surfactant ratio was critical but did not significantly (p > 0.05) affect the CQAs.

The MES study identified five out of the proposed six process parameters as statistically significant CPPs. Polymer surfactant ratio was found to be important but not a significant process parameter. The mathematical expressions for the effect of each CPP on CQAs are represented below:

(2) $$PS=101.428+0.738\left(\frac{\left(U-11\right)}{9}\right)-0.837\left(\frac{\left(V-7.5\right)}{2.5}\right)\;-15.008\left(\frac{\left(W-5.5\right)}{2.5}\right)+X+Y+Z$$

(3) $$PSD=2.093+0.253\left(\frac{\left(U-11\right)}{9}\right)+0.058\left(\frac{\left(V-7.5\right)}{2.5}\right)\;+0.091\left(\frac{\left(W-5.5\right)}{2.5}\right)+X+Y+Z$$

(4) $$EE=14.919-8.604\left(\frac{\left(U-11\right)}{9}\right)+0.401\left(\frac{\left(V-7.5\right)}{2.5}\right)\;+5.391\left(\frac{\left(W-5.5\right)}{2.5}\right)+X+Y+Z$$

(5) $$RT=4.417+2.416\left(\frac{\left(U-11\right)}{9}\right)+0.25\left(\frac{\left(V-7.5\right)}{2.5}\right)\;-0.75\left(\frac{\left(W-5.5\right)}{2.5}\right)+X+Y+Z$$

where, PS = Particle size, PSD = particle size distribution, EE = entrapment efficiency, RT = release time, U = polymer: drug ratio, V = polymer: surfactant ratio, W = dispersion quantity, X = dispersion (silicon oil) viscosity (X_1_ = (10 cSt, X_2_ = 20 cSt, or X_3_ = 100 cSt), Y = sonication time, (Y_1_ = 30 min or 60 min = Y_2_), and sonication energy = Z (Z_1_ + 25% amplitude, Z_2_ = 50% amplitude, or Z_3_ = 75% amplitude).

The values of X, Y, and Z are indicated at three levels represented by subscripts 1, 2, or 3 as shown below for each of the CPPs:

For PS:

X_1_ = 3.898, X_2_ = −8.034, or X_3_ = 4.136,

Y_1_ = 12.772 or Y_2_ = −12.77_2_

Z_1_ = 13.248, Z_2_ = −18.027, or Z_3_ = 4.778

For PSD:

X_1_ = −0.569, X_2_ = 0.174, or X_3_ = 0.395

Y_1_ = −0.13 or Y_2_ = 0.13

Z_1_ = −0.084, Z_2_ = −176, or Z_3_ = 0.261

For EE:

X_1_ = −1.962, X_2_ = 5.26, or X_3_ = −3.297,

Y_1_ = 0.498 or Y_2_ = −0.498

Z_1_ = −0.437, Z_2_ = −2.636, or Z_3_ = 3.073

For RT:

X_1_ = −1.667, X_2_ = 1.8, or X_3_ = −0.133

Y_1_ = 1.417 or Y_2_ = −1.417

Z_1_ = 0.867, Z_2_ = 1, or Z_3_ = −1.867

Sonication time (p < 0.01), sonication energy (p < 0.05), and dispersion medium quantity (p < 0.05) were the significant CPPs influencing particle size. It was interesting to note that polymer-drug ratio, and dispersion medium viscosity were not statistically significant in affecting the particle size of microparticles individually. Although, there are evidence that particle size is affected by the polymer concentration [71], and dispersion medium viscosity [72], they were not found to be significant. The dynamic interplay of several CPPs at the same time could be a possible reason for such an observation. This phenomenon is further discussed below in section titled “Response Surface”, where we observed the secondary interactions of polymer-drug ratio with other factors. Hence, a process parameter may not be significant on its own but the simultaneous interaction with another parameter may render a significant effect. With increasing sonication time and energy, the particle size showed a decreasing trend. This observation is obvious considering higher energy input in the coacervation process prevents the formation of polymer aggregates [73].

However, MES study outcome was interesting which indicated that the particle size distribution was significantly affected by polymer-drug ratio (p < 0.05) and the dispersion medium viscosity (p < 0.05). These finding align with prior literature on how polymer concentration impacts the particle characteristics. Elevating the polymer-to-drug ratio activates the particle nucleation process, which, in turn, serves as a fundamental contributor to particle aggregation and the emergence of a wider size distribution [74]. Evidence also suggests that higher dispersion medium viscosity leads to the production of larger particles [75–78], along with a wider size distribution.

Lastly, both the polymer drug ratio (p < 0.01) and the sonication time (p < 0.05) were significant CPPs for drug release duration. Higher polymer concentration leads to slower degradation of the polymeric microparticle and hence a slower release of the drug, leading to sustained release. On the other hand, higher sonication time leads to small particle sizes which have a faster release rate or shorter release duration as compared to larger particles due to their relatively greater polymer content/concentration.

### Response Surface Design (RSD)

**Fig. 3:**
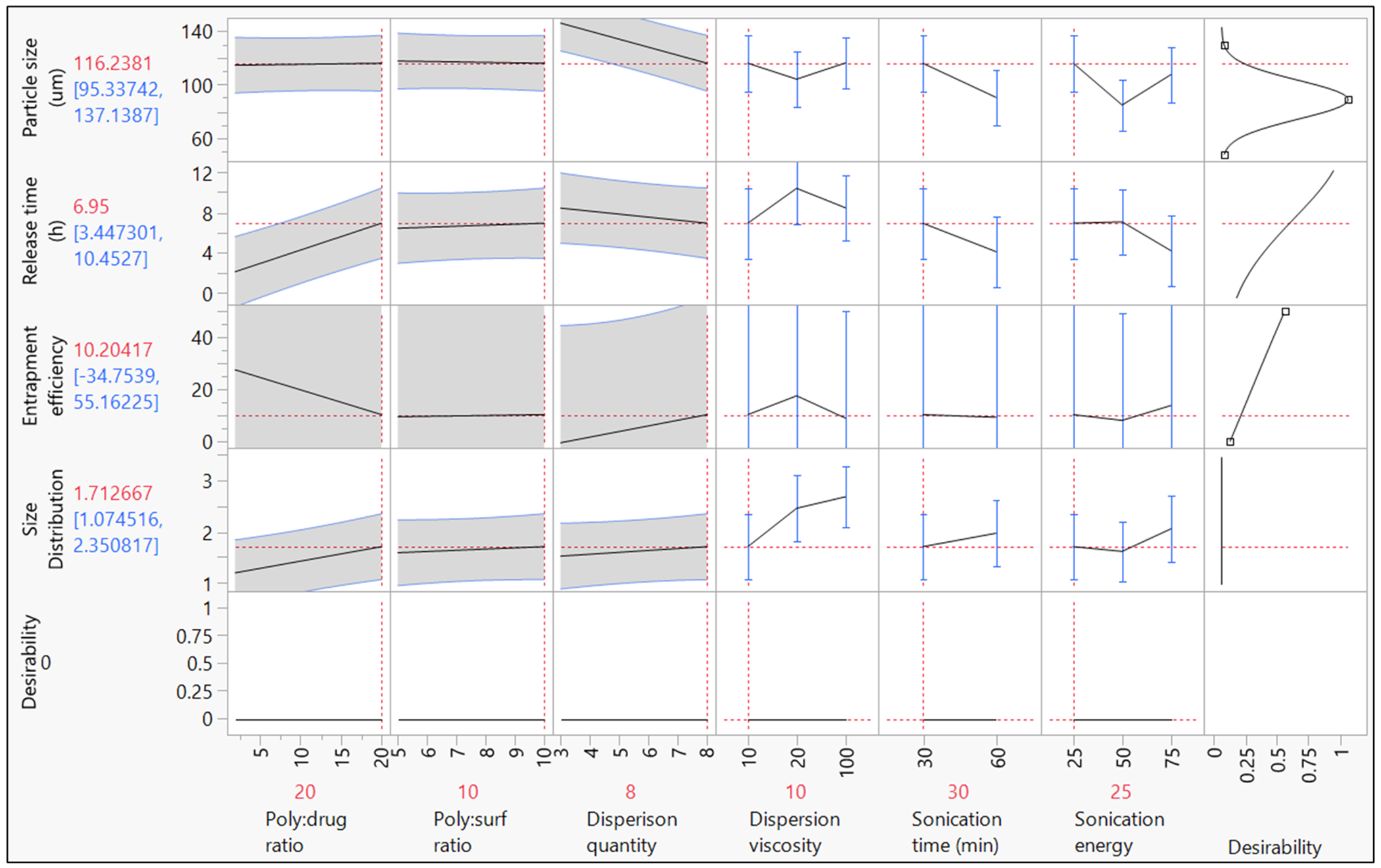
Optimal values of CPPs by simultaneous dynamic Optimization of CQAs using prediction profiler

Using the dynamics prediction profiler feature in JMP, all the parameters were optimized for maximum simultaneous desirability of the four CQAs as shown in Fig. 3. This was achieved using the “Maximize Desirability” feature of JMP post-analysis of the model. All the CQAs (responses) were given equal importance while designing the MES and RSD models which affects how JMP determines maximum desirability. The model returned the values of CPPs that led to the best possible trade-off values of the CQAs. For the non-significant process parameter, the polymer surfactant ratio of 10 was fixed. The RSD model cannot accommodate categorical variables: hence dispersion medium viscosity was fixed based on MES model to 20 cSt. For the other significant CPPs, the same value ranges used for MES were used for optimization in the RSD model.

The RSD recommended 27 formulation trials (Table IV). These points are a combination of factorial points, axial points, and centre points. Using the parameters for CPPs described in Table IV, formulations were prepared for a constant drug load of 50 mg and characterized for particle size, entrapment efficiency, and release time using the method described in Methods section*.* Table V summarizes the data obtained from the characterization studies.

When the experimental outcomes were input into the RSD model and analyzed using the least squares method, a detailed report on the effect of the CPPs on the CQAs was obtained. The RSD identified the polymer-drug ratio (p < 0.05), sonication time (p < 0.05), and sonication energy (p < 0.05) (Table I supplementary) and the secondary interactions of polymer-drug ratio as significant (p < 0.05) CPPs (Table II supplementary). The secondary interaction of polymer-drug ratio indicates a higher degree of quadratic relationship between the CPP and CQAs indicating a parabolic trend. Therefore, a maximum value of polymer-drug ratio could be optimal for maximizing the CQAs. Interestingly, the dispersion medium quantity did not elicit a significant effect on the CQAs, although the effect was critical. The quantity of dispersion medium is important in determining the “stability window” of the microparticles. Until the desired concentration of dispersion quantity is reached, the particles formed are prone to aggregation and fusion [79]. Hence, the quantity of silicone oil determines both the particle size and particle size stability [61].

In the RSD, secondary interactions between the CPPs were analyzed along with the effect of individual CPPs. The particle size was significantly affected by the sonication energy (p < 0.05) as also observed in MES. From the prediction profiler, it is evident that the RSD described a quadratic relation between particle size and polymer drug ratio, in contrast to the linear relationship predicted by MES. The particle size did not show a significant relationship with the polymer concentration and dispersion medium quantity. The presence of an intercept and block term indicates that in the overall process design and the properties of formulation components, there could be unidentified or inherent factors that affect the particle size more than the CPPs studied here. The particle size distribution and drug release time also did not have a statistically significant relationship with all four CPPs evaluated in the RSD. However, the effect of each CPP on both particle size distribution and release time can be quantified using the numerical equations listed below:

(6) $$PS=99.934+3.526\left(\frac{\left(U-11\right)}{9}\right)-17.408\left(\frac{\left(W-5.5\right)}{2.5}\right)\;+4.939\left(\frac{\left(Y-45\right)}{15}\right)+24.307\left(\frac{\left(Z-50\right)}{25}\right)\;+\left(\frac{\left(U-11\right)}{9}\right)\left(\left(\frac{\left(W-5.5\right)}{2.5}\right)(-34.048)\right)\;+\left(\frac{\left(U-11\right)}{9}\right)\left(\left(\frac{\left(U-11\right)}{9}\right)(-16.431)\right)+B$$

(7) $$\mathrm{PSD}=1.139-0.147\left(\frac{\left(U-11\right)}{9}\right)+0.003\left(\frac{\left(W-5.5\right)}{2.5}\right)\;+0.125\left(\frac{\left(Y-45\right)}{15}\right)+0.180\left(\frac{\left(Z-50\right)}{25}\right)\;+\left(\frac{\left(U-11\right)}{9}\right)\left(\left(\frac{\left(W-5.5\right)}{2.5}\right)0.008\right)\;+\left(\frac{\left(U-11\right)}{9}\right)\left(\left(\frac{\left(U-11\right)}{9}\right)(-0.301)\right)+B$$

(8) $$EE=3.206-3.215\left(\frac{\left(U-11\right)}{9}\right)+0.504\left(\frac{\left(W-5.5\right)}{2.5}\right)\;-2433\left(\frac{\left(Y-45\right)}{15}\right)-0.353\left(\frac{\left(Z-50\right)}{25}\right)\;+\left(\frac{\left(U-11\right)}{9}\right)\left(\left(\frac{\left(W-5.5\right)}{2.5}\right)6.969\right)\;+\left(\frac{\left(U-11\right)}{9}\right)\left(\left(\frac{\left(U-11\right)}{9}\right)5.614\right)+B$$

(9) $$RT=3.6-0.5\left(\frac{\left(U-11\right)}{9}\right)+0.75\left(\frac{\left(W-5.5\right)}{2.5}\right)-0.5\left(\frac{\left(Y-45\right)}{15}\right)\;+24.31\left(\frac{\left(Z-50\right)}{25}\right)+\left(\frac{\left(U-11\right)}{9}\right)\left(\left(\frac{\left(W-5.5\right)}{2.5}\right)(-0.75)\right)\;+\left(\frac{\left(U-11\right)}{9}\right)\left(\left(\frac{\left(U-11\right)}{9}\right)0.233\right)+B$$

where, B = Block of CPPs which would have values at three levels represented by subscripts 1, 2, or 3 as shown below for each of the CQAs:

For PS:

B_1_ = −1.33, B_2_ = −22.95, or B_3_ = 24.28

For PSD:

B_1_ = 0.15, B_2_ = − 0.10 or, B_3_ = −0.05

For EE:

B_1_ = 1.71, B_2_ = −1.75, or B_3_ = 0.04

For RT:

B_1_ = −0.04, B_2_ = 0.07, or B_3_ = −0.04

The entrapment efficiency of the microparticles was significantly affected by several factors in the RSD model. Firstly, polymer-drug ratio had a relationship with the entrapment efficiency (p < 0.05) as a primary CPP. Moreover, a quadratic relationship is also observed due to interaction of polymer-drug ratio with itself, and its interaction with dispersion medium quantity (p < 0.005). Consequently, the model indicates that when it comes to the microparticle preparation process employed in our study, it is important to consider the intricate dynamics between polymer concentration and the quantity of dispersion medium. The compounded effect of these two CPPs affect the entrapment efficiency more significantly than the individual parameters themselves. Lastly, increasing the sonication time led to a significant decrease in the entrapment efficiency. Interestingly, we also observed a trade-off between the particle size and entrapment efficiency with increase in the sonication time, which is consistent with the literature reports [80]. Medium size particles, obtained with shorter sonication times, provided a better entrapment efficiencies as larger sonication periods can potentially lead to breakdown of the microparticles being formed.

**Fig. 4:**
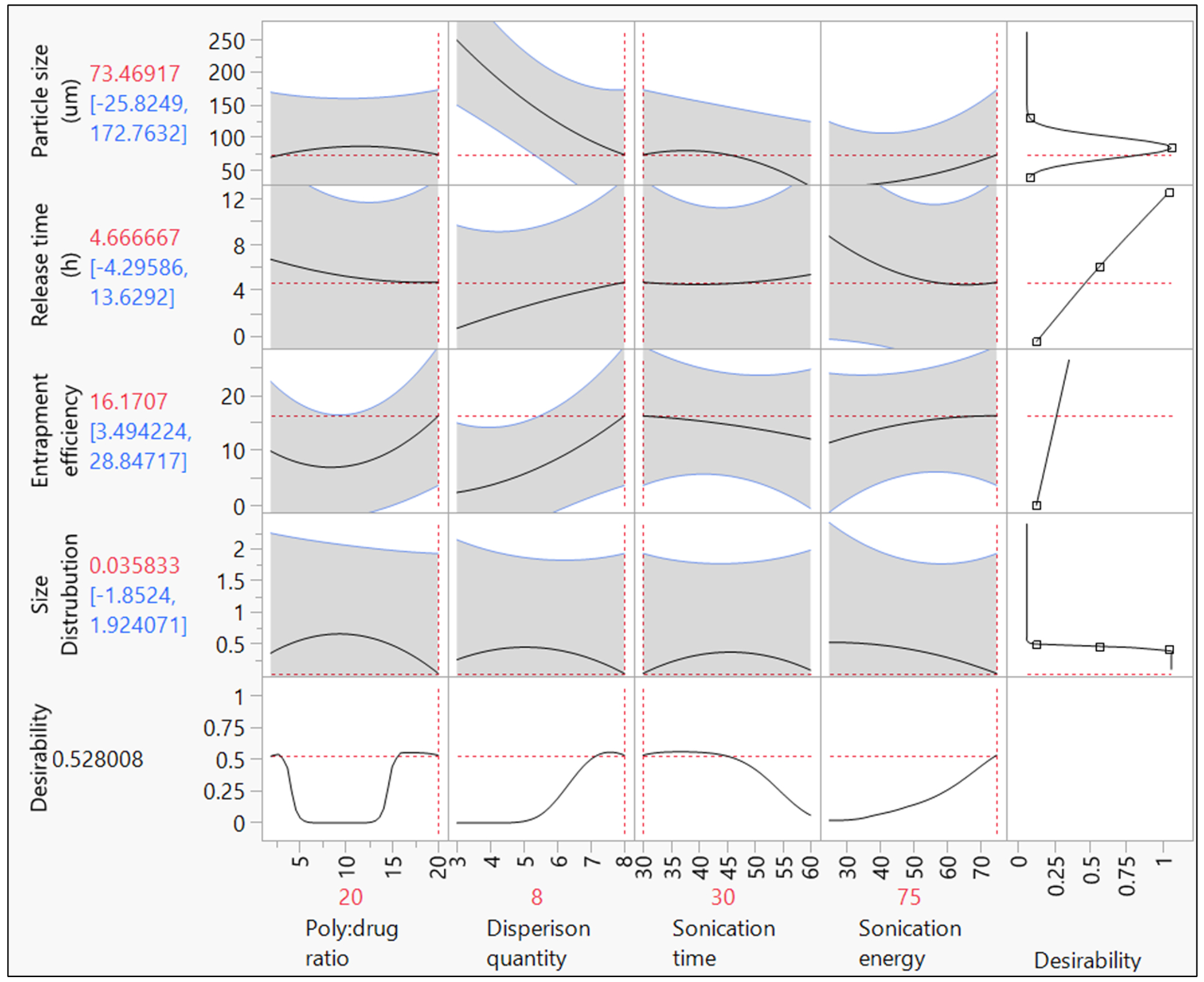
Prediction profiler which can dynamically optimize the CQAs simultaneously and return the optimum values of CPPs

In summary, all the CPPs identified in the RSD showed a statistically significant primary and/or secondary effect on the CQAs. As represented in the prediction profiler Fig. 4 a non-linear quadratic relationship was observed. This relationship can also be understood by surface plots shown in Fig. 1 supplementary. Using the dynamics prediction profiler feature in JMP, all the parameters were optimized for maximum simultaneous desirability of the four CQAs. According to the model, polymer-drug ratio of 20, dispersion medium quantity of 8 g. sonication time of 40 min and sonication energy of 70 amplitude exhibited the maximum desirability for all three factors considered simultaneously with the primary and secondary interactions of the defined parameters. Such an experimental analysis cannot be obtained from the classical one-factor-at-a-time (OFAT) approach. A design including factorial, axial, and star points can help in understanding the effect of parameters at higher levels. Using the RSD model, a quantitative relationship between the effect of two significant CPPs on the overall product as well as individual CQAs was established.

During the screening, polymer-surfactant ratio, polymer to drug ratio, and amount of dispersion medium were kept as continuous factors because we were not knowing what their optimal values could be for obtaining an extended release by using specific categorical values for silicon oil viscosity, sonication energy, and sonication time. Once JMP provided optimal values of continuous parameters, their specific categorical values were used later on while continuously changing duration and energy levels of sonication to optimize the final formulation.

### Validation of RSD

As per the ICH guidelines [81], the robustness of a QbD model is its ability to demonstrate acceptable quality and performance while tolerating variability in inputs, which can be a function of both formulation and process design. Hence, to validate the responses generated by the RSD model, optimized CPPs were used to formulate microparticles. These microparticles were characterized by adding the CQAs values obtained experimentally and were compared to those predicted by the RSD model (Table VI). The percent deviation of the actual experimental value from the model-predicted value was calculated using the formula:

(10) $$\%\mathrm{Deviation}=\frac{\mathrm{Predicted value}-\mathrm{Experimental value}}{\mathrm{Experimental value}}$$

As represented in Table VI, the experimental values were in reasonable agreement and more favorable than the predicted values. The experimental particle size had a 33.28% deviation with the predicted particle size. However, the experimental value is more favourable over the predicted value since the limit of particle size of syringeability is 159 μm for a relatively greater patient compliant using needle size of 30G. Hence, the particle size, considering the particle size distribution, should be as high as possible but not exceeding the limit of syringeability (159 μm for 30G, 254 μm for 25G) [58] to achieve an optimum drug load. Therefore, the experimental value of particle size is superior to the predicted value. Similarly, the drug release time found experimentally was longer than the predicted value. A sustained release time is desired as it will decrease the application frequency of the formulation. This is highly favourable in our scenario for a subconjunctival dosage form.

Moreover, the experimental value for entrapment efficiency was also superior to that of predicted value. Again, this is beneficial since more % entrapment ensures adequate drug loading and in turn smaller formulation dose. Lastly, the size distribution of particles was also higher than the predicted value. A lower size distribution ranges is desired since a smaller size distribution will lead to uniformity in the particle size. However, both the predicted and experimental values comply with the generally accepted values for polymeric microparticles.

## Conclusions

In conclusion, QbD helped in identifying, optimizing, and quantifying the effect of process parameters of the microparticles and predicting a set of parameters for maximum desirability of all four CQAs. However, the particle properties obtained in this study still need to be optimized in multiple aspects. For example, ideally the entrapment efficiency should be higher than 70%, which was not observed here. Since the process needs to be devoid of water, which is detrimental for HSD, simpler approaches such as adjusting the continuous phase are not applicable here [82]. Nevertheless, other strategies such as using high molecular weight polymer [83], using a more hydrophobic polymer and using a co-polymer blend for optimal hydrophobicity [84] could be potentially useful. The drug release duration was also not sufficient from the context of an ocular injectable dosage form. The release should last up to several days instead of hours. Interestingly, the same strategies to improve the entrapment efficiency, namely using high molecular weight polymer, and using a more hydrophobic polymer, could potentially also lead to a higher release duration. It must be noted that for all experiments in this study, the maximum aqueous content of H_2_S was lower than its reported solubility in water, which is 3.9 g/L (120 mM) at 20 °C [85]. The solubility of H_2_S is critically dependent on temperature and the solubility decreases with increasing temperature [86]. The release study was performed at 37 °C which might have resulted in the loss of H_2_S which can be minimized by conducting such study at lower temperatures e.g., 10 °C (solubility of 5.3 g/L) or 0 °C (solubility of 7.1 g/L). Hence, the future prospective of the formulation of these polymeric microparticles is utilizing one or more of the above approaches including higher drug load to achieve a particle system that sustains the release of H_2_S for longer periods of time while complying with the particle size, entrapment efficiency and size distribution requirements. Some strategies that we have been working on are using a slow releasing HSD such as GYY4137, using an *in situ* gelling dual polymer system, using slower release polymers such as polyanhydrides, or the combination of multiple strategies including cross-linking agents known to decrease diffusivity [87, 88]. The goal, however, is to extend the release from the current outcome of hours to days and weeks. This sustained-release delivery system should be able to provide safe and therapeutic doses of H_2_S for longer periods of time. Such an ideal delivery system of H_2_S would be an important development in the management of glaucoma.

## Supplementary Material

Fig. 1. supplementary. Surface plot depicting the nature of relationship between different CQAs and CPPs

Table I supplementary Cumulative effect of CPPs on CQAs of microparticles from RSD model represented as p-values

Table II supplementary Effect of secondary interactions of CPPs on CQAs of microparticles using RSD model represented as p-values

The online version contains supplementary material available at https://doi.org/10.1208/s12249-024-02840-8.

## Figures and Tables

**Table I:** Main Effects Screening Design Generated by JMP with 12 Trials

Trial #	Polymer:drug ratio (w/w)	Polymer: surfactant ratio (w/w)	Dispersion quantity (g)	Dispersion viscosity (cST)	Sonication time (min)	Sonication energy* (% amplitude)
1	2	5	3	10	60	50
2	2	10	8	10	60	25
3	20	5	8	20	60	50
4	2	10	3	100	30	50
5	20	5	3	10	30	75
6	20	10	8	100	60	75
7	20	5	3	100	60	25
8	2	5	8	100	30	25
9	20	10	8	10	30	50
10	2	10	3	20	60	75
11	2	5	8	20	30	75
12	20	10	3	20	30	25

* Sonicator probe diameter = 0.24 inch and maximum amplitude = 200 μm

**Table II:** Experimental values for the four CQAs from the MES experimental design

Trial number	Particle size (μm)	Particle size distribution	Release time (h)	Entrapment efficiency (%)
1	90.02	1.07	0	4.23
2	87.01	1.56	0	36.16
3	51.53	2.63	8	15.27
4	111.53	1.99	6	22.84
5	141.82	1.88	6	3.97
6	82.90	3.35	3	6.84
7	117.55	2.47	6	4.88
8	123.51	2.06	3	11.49
9	84.42	1.41	6	4.83
10	102.92	2.26	1	19.15
11	89.14	2.10	2	47.27
12	134.76	2.34	12	2.10

**Table III:** Cumulative Effect of CPPs on CQAs of Microparticles Based on MES Design Represented Using p Values

Factors	Overall	Particle size	Particle size distribution	Entrapment efficiency	Drug release
Polymer: drug ratio	0.0063**	0.7483	0.0288*	0.1526	0.0063**
Polymer: surfactant ratio	0.4296	0.7165	0.4296	0.9348	0.5283
Dispersion medium quantity (g)	0.0056**	0.0056**	0.2478	0.3181	0.1226
Dispersion medium viscosity (cST)	0.0192*	0.1674	0.0192*	0.745	0.0666
Sonication time (min)	0.0089**	0.0089**	0.1354	0.9192	0.0275*
Sonication energy	0.0204*	0.0204*	0.1409	0.885	0.0794

* Indicates significance at p<0.05,

** at p<0.01, and

*** at p<0.005

**Table IV:** Box Behnken Response Surface Design Generated by JMP with 27 Trials

Trial	Pattern*	Block	Poly:drug ratio (w/w)	Dispersion quantity (g)	Sonication time (min)	Sonication energy (% amplitude)
1	+−00	1	20	3	45	50
2	0	1	11	5.5	45	50
3	00+−	1	11	5.5	60	25
4	−+00	1	2	8	45	50
5	−−00	1	2	3	45	50
6	00−−	1	11	5.5	30	25
7	00−+	1	11	5.5	30	75
8	++00	1	20	8	45	50
9	00++	1	11	5.5	60	75
10	−00−	2	2	5.5	45	25
11	0+−0	2	11	8	30	50
12	−00+	2	2	5.5	45	75
13	0−+0	2	11	3	60	50
14	+00−	2	20	5.5	45	25
15	0−−0	2	11	3	30	50
16	0++0	2	11	8	60	50
17	+00+	2	20	5.5	45	75
18	0	2	11	5.5	45	50
19	0−0+	3	11	3	45	75
20	0+0+	3	11	8	45	75
21	0	3	11	5.5	45	50
22	+0+0	3	20	5.5	60	50
23	−0+0	3	2	5.5	60	50
24	0−0−	3	11	3	45	25
25	−0−0	3	2	5.5	30	50
26	0+0−	3	11	8	45	25
27	+0−0	3	20	5.5	30	50

* represents high level,—represents low level, and 0 represents center

**Table V:** Experimental Values for the Four CQAs from the MES Experimental Design

Trial	Particle size (μm)	Release time (h)	Entrapment efficiency (%)	Particle size distribution
1	166.01	1	0.88	0.51
2	103.22	4	1.17	2.68
3	69.21	2	1.34	1.27
4	101.15	6	11.39	1.10
5	83.57	0	26.00	0.66
6	47.07	12	1.34	0.92
7	123.92	2	8.71	0.78
8	47.39	4	14.16	0.98
9	80.16	2	1.68	1.50
10	61.84	4	12.36	1.01
11	60.22	2	4.86	1.07
12	54.77	8	4.81	1.29
13	56.66	4	1.68	0.95
14	54.78	4	2.36	0.54
15	69.49	2	2.18	0.71
16	78.69	4	2.68	0.64
17	95.22	4	2.65	0.64
18	95.41	2	2.01	1.30
19	257.87	2	2.51	2.10
20	141.09	6	2.51	1.07
21	87.50	2	5.70	0.61
22	77.65	1	4.42	0.87
23	128.60	4	1.44	1.10
24	116.13	6	3.02	0.68
25	49.90	4	16.21	0.75
26	112.30	2	6.70	0.80
27	81.10	6	9.14	0.61

**Table VI:** Validation of RSD Model with Experimental Results

Parameter	RSD Predicted	Experimental	% Deviation
Particle size (μm)	53.37	80 ± 16	33.28
Release time (hours)	3.23	3.5 ± 0.76	7.71
Encapsulation efficiency (%)	13.61	17.24 ± 5.15	21.11
Particle size distribution	0.80	0.997 ± 0.04	19.79
