# Supplementary figures and images for: Application of Quality by Design in the Development of Hydrogen Sulfide Donor Loaded Polymeric Microparticles
Source: AAPS PharmSciTech. Author manuscript; Available in PMC 2026 Jul 8. (PMC13344383; doi:10.1208/s12249-024-02840-8)

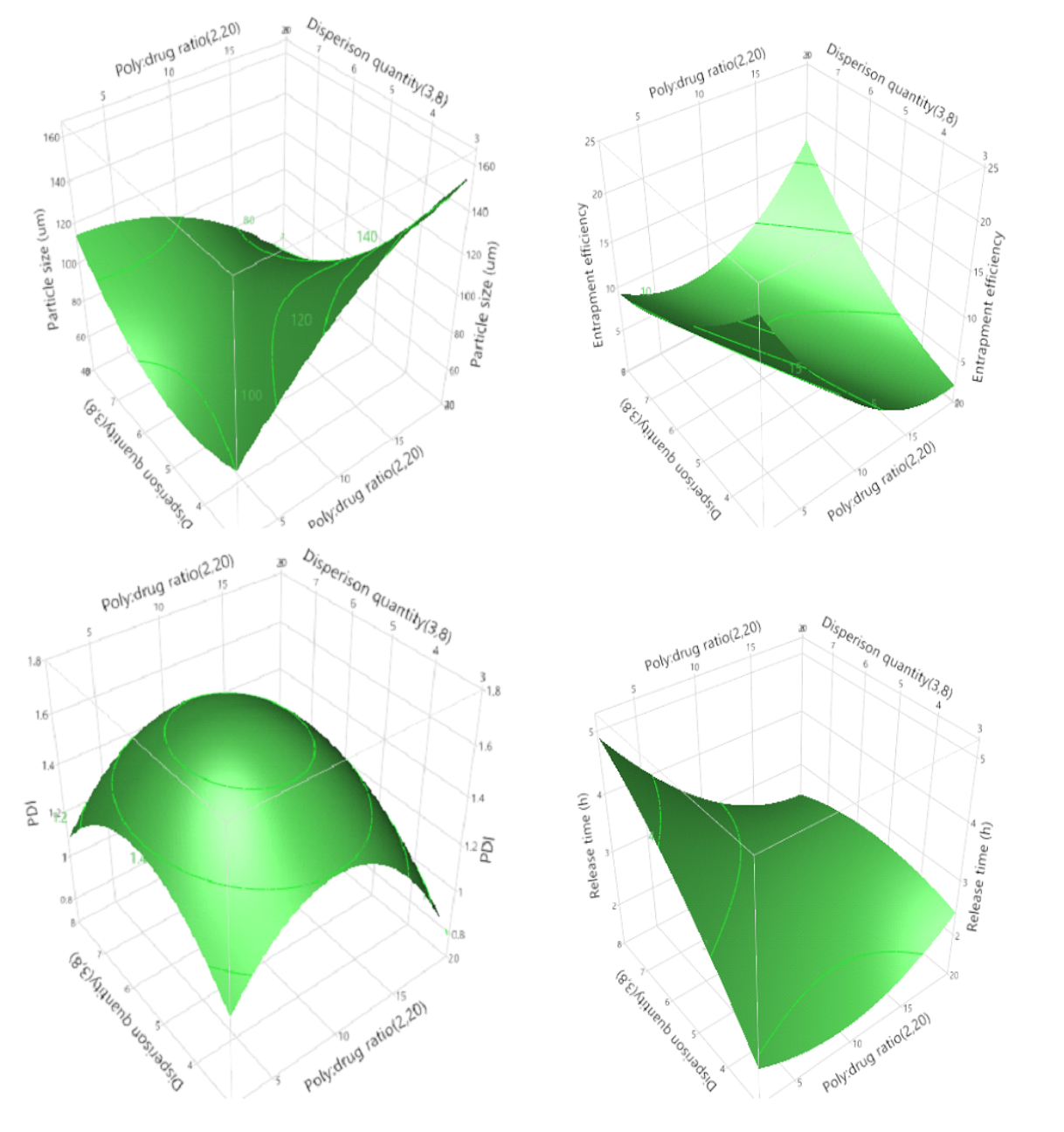

Supplement: Fig. 1. supplementary. Surface plot depicting the nature of relationship between different CQAs and CPPs [file NIHMS2184549-supplement-Fig__1__supplementary__Surface_plot_depicting_the_nature_of_relationship_between_different_CQAs_and_CPPs.png]
