# Supplementary material for: Application of Quality by Design in the Development of Hydrogen Sulfide Donor Loaded Polymeric Microparticles
Source: AAPS PharmSciTech. Author manuscript; Available in PMC 2026 Jul 8. (PMC13344383; doi:10.1208/s12249-024-02840-8)
Supplement: Table I supplementary Cumulative effect of CPPs on CQAs of microparticles from RSD model represented as p-values [file NIHMS2184549-supplement-Table_I_supplementary___Cumulative_effect_of_CPPs_on_CQAs_of_microparticles_from_RSD_model_represented_as__p-values.docx]

**Table I supplementary** Cumulative effect of CPPs on CQAs of microparticles from RSD model represented as p-values

| Factors | Overall | Particle size | Particle size distribution | Entrapment efficiency | Drug release |
| --- | --- | --- | --- | --- | --- |
| Polymer: drug ratio | 0.0082* | 0.7458 | 0.5396 | 0.0082* | 0.3121 |
| Dispersion quantity (g) | 0.1215 | 0.1215 | 0.3607 | 0.6474 | 0.9814 |
| Sonication time (min) | 0.0373* | 0.6503 | 0.2667 | 0.0374* | 0.3889 |
| Sonication energy | 0.0358* | 0.0358* | 0.5396 | 0.7484 | 0.2175 |

*^*^ Indicates significance at p<0.05, ^**^ at p<0.01, and ^***^ at p<0.005*
