# Supplementary material for: Application of Quality by Design in the Development of Hydrogen Sulfide Donor Loaded Polymeric Microparticles
Source: AAPS PharmSciTech. Author manuscript; Available in PMC 2026 Jul 8. (PMC13344383; doi:10.1208/s12249-024-02840-8)
Supplement: Table II supplementary Effect of secondary interactions of CPPs on CQAs of microparticles using RSD model represented as p-values [file NIHMS2184549-supplement-Table_II_supplementary___Effect_of_secondary_interactions_of_CPPs_on_CQAs_of_microparticles_using_RSD_model_represented_as_p-values.docx]

**Table II supplementary** Effect of secondary interactions of CPPs on CQAs of microparticles using RSD model represented as p-values

| Factors | Overall | Particle size | Particle size distribution | Entrapment efficiency | Drug release |
| --- | --- | --- | --- | --- | --- |
| Polymer: drug ratio*dispersion quantity | 0.0016*** | 0.0831 | 0.5948 | 0.0016*** | 0.9759 |
| Polymer: drug ratio*polymer: drug ratio | 0.0011*** | 0.2679 | 0.8303 | 0.0011*** | 0.1288 |
| Block | 0.0466* | 0.0466 | 0.9952 | 0.1770 | 0.5273 |

*^*^ Indicates significance at p<0.05, ^**^ at p<0.01, and ^***^ at p<0.005*
